# 2187: Investigation of antimicrobial resistance in Ureaplasma species and Mycoplasma hominis isolates from urine cultures in college-aged females

**DOI:** 10.1017/cts.2017.98

**Published:** 2018-05-10

**Authors:** Marissa Valentine-King, Mary B. Brown

## Abstract

OBJECTIVES/SPECIFIC AIMS: Urinary tract infections (UTIs) serve as one of the most common infections affecting women. With rising reports of antibiotic resistance (ABR), which can prolong illness and limit treatment options, the Infectious Disease Society of America recommends using local resistance patterns to shape empirical treatment selection. Although no studies have evaluated ABR in Ureaplasma spp. urinary isolates in college-aged women, regional studies in the Southeast United States have found levels of tetracycline resistance in over 30% of Ureaplasma spp. clinical isolates. Thus, this study aims to determine the antibiogram for 73 Ureaplasma spp. and 10 Mycoplasma hominis isolates collected from women with first-time UTI against a panel of 9 antibiotics, and assess resistant isolates for genetic mechanisms associated with resistance. METHODS/STUDY POPULATION: This study used archival samples and data collected from college-aged women with first-time UTI recruited to participate in a prospective cohort study conducted at a student healthcare facility from 2001 to 2006 in Florida. Ureaplasma spp. and M. hominis isolates cultured from urine samples collected at the initial clinical presentation and for any recurrent UTI were evaluated for susceptibility to a panel of 9 antibiotics (8 for M. hominis) using validated microbroth and agar dilution methods, respectively. Ureaplasma spp. isolates were tested against azithromycin, chloramphenicol, ciprofloxacin, clindamycin, erythromycin, doxycycline, gentamicin, levofloxacin, and tetracycline. M. hominis isolates underwent the same testing, with the addition of linezolid and exclusion of azithromycin and erythromycin, as M. hominis is intrinsically resistance to 14 and 15-membered macrolides and azilides. PCR and Sanger sequencing were employed to identify molecular mechanisms associated with resistance. RESULTS/ANTICIPATED RESULTS: Of the 73 Ureaplasma spp. isolates, 1 isolate was resistant to levofloxacin (MIC: 4 µg/mL) and 1 to tetracycline (MIC: 8 µg/mL). All M. hominis isolates were sensitive. For the Ureaplasma spp. isolates, MIC90s were highest against gentamicin (32 µg/mL) and lowest against doxycycline (0.25 µg/mL). PCR amplification identified tetM present in the tetracycline resistant isolate, an established gene associated with tetracycline resistance in Ureaplasma spp. A S83W mutation within the quinolone-resistance-determining region (QRDR) of parC was detected in the levofloxacin resistant isolate. DISCUSSION/SIGNIFICANCE OF IMPACT: Overall, antibiotic resistance in this population of college-aged women with first-time UTI was low. A previous study detected a novel S83W substitution in a perinatal Ureaplasma spp. isolate from Japan, and provided in silico evidence that a S83W change would prevent levofloxacin from binding to its target. However, that study was unable to cultivate the isolate. Our study has provided the corresponding phenotypic evidence that a S83W substitution results in quinolone resistance in Ureaplasma spp.

